# Advancing women in science: closing the final gap

**DOI:** 10.1016/j.lana.2024.100840

**Published:** 2024-07-15

**Authors:** The Lancet Regional Health – Americas

Women's participation in research reached the parity zone for the first time in 2022, according to the new Elsevier report on Gender and Diversity in Research released in June 2024, showing 41% of researchers are women. While this is undoubtedly a significant milestone, these numbers drop to 27% for women in advanced career stages. The retention of women in research and the underrepresentation of women in leadership positions remain critical challenges to achieving true gender equality in science.

In 2022, Knaul and colleagues wrote about the harms of the continued loss of women in medicine and research in Latin America, where they represent the majority of medical doctors, but that this majority is not reflected in the number of appointed members of national academies or leadership positions. Deans, presidents and endowed professors continue to be majoritarian men. Supporting the argument that more does not necessarily translate into merrier, the authors highlight the need for a systemic change to ensure women are not only equally participating in science but leading it, thriving, and being properly recognised.

The barriers encountered by young researchers, which impede or postpone their career progression, are neither new nor unfamiliar. These obstacles encompass a range of barriers such as implicit bias in evaluation and promotion processes, invisible contribution, like when a male colleague is perceived as co-author and a female is just helping out the group by doing the same tasks, a dearth of leadership models and mentors, and imbalanced societal expectations regarding traditional gender roles, among others. While some of these barriers are still unfairly challenged as subjective, supporting data have shown that women are less likely to secure research grants, be named in a given article, and receive fewer citations than men. One interesting finding from Elsevier's latest report was that women tend to be more cited in policy documents. This finding is not surprising as women also compose the majority of researchers in SDG-related fields, a reflection of structural patriarchal roots in the system and traditional roles of women in care-related positions.

The challenges faced by women in science are further compounded by intersectional barriers faced by Black, Hispanic and Indigenous women. In the US, Black and Hispanic women in Science, Technology, Engineering, and Mathematics (STEM) jobs earn about $20,000 a year less than the average for the same job and about $33,000 less than their white male counterparts. For Latin American researchers, the local barriers compound an additional dose of structural machismo. When the internal barriers are overcome, the few women who rise to the top are much less likely to receive international recognition. Representation of women from minoritised races and ethnicities in research output, editorial boards, grant panels and as conference speakers is even lower, a striking reminder of the racist roots of medical sciences.

Breaking this cycle is not easy. We have made tremendous advances in including more women and girls in science and medicine over the last decades but the next challenge, to foster their advance to leadership positions and establish a generation of diverse models and mentors is a much more difficult one. Science funding agencies have increasingly recognised the importance of a diverse research environment and the need to increase support to advance women in research. Designated grants, fellowships and awards for early-career female researchers have been introduced by most of the major funders, including in Brazil, Mexico and the US. In the Netherlands, a radical women-only hiring policy helped the Eindhoven University of Technology increase diversity. Those are important movements that can have impactful reparatory consequences; however, a systemic change is the only pathway that can lead to sustained improvement. We need the entire system to work on an equal level. We need women to choose a career in research because they know they will compete on an equal basis for a grant and that their work will be recognised solely for its scientific impact.

The ‘*system*’ is composed of a complex combination of players, equally responsible for advancing this agenda. In 2019, *The Lancet* launched a special themed issue on “Advancing women in science, medicine, and global health” that kicked off an internal commitment to advocate for and advance gender equality. Since then, The Lancet group has embraced several editorial commitments, including a no all-male panel policy and an internal endeavour to achieve gender balance across editorial boards and reviewer pools, and actively working towards increasing representation in authorship.

In line with our pledge to promote gender equality in science and recognising that Latinx researchers face intersectional barriers in the advancement of their careers, *The Lancet Regional Health – Americas* editorial team is thrilled to introduce our new project “*Latinas in Research: stories of strength, commitment and perseverance”,* aimed at increasing the visibility of remarkable female researchers from Latin America. Over the next 6 months, our journal will feature a profile of one outstanding researcher in each issue, delving into their career trajectory, achievements and challenges.

By amplifying the voices of these exceptional women, we hope to inspire future generations of female scientists and foster a more inclusive scientific community. We firmly believe that their stories will serve as a catalyst for change, urging us all to address the systemic issues that continue to hinder the progress of women in science.

We understand that achieving true gender parity in science requires simultaneous changes at both the individual and institutional levels and invite all our readers to join us on this journey.

